# Good-Night (Poetry)

**Published:** 1876-06

**Authors:** 

**Affiliations:** St. Nicholas


### *Good Night.*

What do I see in Baby's eyes?
So bright! so bright!
I see the blue, I see a spark,
I see a twinkle in the dark—
Now shut them tight.

What do I see in Baby's eyes?
Shut tight—shut tight.
The blue is gone, the light is hid—
I'll lay a soft kiss on each lid.
Good night! good night!

—*Mrs. Dodge, St. Nicholas for June.*

